# *Oenococcus oeni* Lifestyle Modulates Wine Volatilome and Malolactic Fermentation Outcome

**DOI:** 10.3389/fmicb.2021.736789

**Published:** 2021-09-28

**Authors:** Rosanna Tofalo, Noemi Battistelli, Giorgia Perpetuini, Luca Valbonetti, Alessio Pio Rossetti, Carlo Perla, Camillo Zulli, Giuseppe Arfelli

**Affiliations:** ^1^Faculty of Bioscience and Technology for Food, Agriculture and Environment, University of Teramo, Teramo, Italy; ^2^Dalton Biotecnologie s.r.l., Spoltore, Italy; ^3^Orsogna Winery, Orsogna, Italy

**Keywords:** *Oenococcus oeni* biofilm, volatilome, malolactic fermentation, wine, Montepulciano d’Abruzzo

## Abstract

In this study, nine *Oenococcus oeni* strains were tested for their ability to adhere to polystyrene using mMRS and wine as culture media. Moreover, planktonic and biofilm-detached cells were investigated for their influence on malic acid degradation kinetics and aroma compound production. Three strains were able to adhere on polystyrene plates in a strain-dependent way. In particular, MALOBACT-T1 and ISO359 strains mainly grew as planktonic cells, while the ISO360 strain was found prevalent in sessile state. The strain-dependent adhesion ability was confirmed by confocal laser scanning microscopy. Planktonic and biofilm detached cells showed a different metabolism. In fact, biofilm-detached cells had a better malic acid degradation kinetic and influenced the aroma composition of resulting wines, acting on the final concentration of esters, higher alcohols, and organic acids. *Oenococcus oeni* in biofilm lifestyle seems to be a suitable tool to improve malolactic fermentation outcome, and to contribute to wine aroma. The industrial-scale application of this strategy should be implemented to develop novel wine styles.

## Introduction

Malolactic fermentation (MLF) consists in the decarboxylation of L-malic acid to L-lactic acid by lactic acid bacteria (LAB)—mainly *Oenococcus oeni*, *Lactiplantibacillus plantarum*, and *Pediococcus* spp. (Capozzi et al., 2021). It occurs at the end of alcoholic fermentation (AF) and results in an enhanced wine flavor and aroma complexity, such as the release of volatile thiols from precursor compounds, methionine metabolism, glycosidase and esterase activities, reduced acidity, and microbial stability (Bartowsky, 2017; Capozzi et al., 2021). The correct outcome of MLF relies on several factors including starter culture concentration and the ability to face the stressful conditions of wine fermentation. Alteration of MLF can cause wine oxidation and/or microbial spoilage (Sumby et al., 2019). *O. oeni* is considered the best-adapted species in wine; it is a Gram-positive, non-motile, facultative anaerobic and chemoorganotrophic bacterium organized in chains or pairs of circular to ellipsoidal cells. It occurs naturally in fruit mashes and related environments and proliferates in wine during or after the AF (Bartowsky, 2017). Several studies recognize *O. oeni* ability to cope with wine stressful conditions, comprising cumulative effects of low pH, high ethanol and SO_2_ content, non-optimal growth temperatures, and growth inhibitory compounds (Bonomo et al., 2018). Some strains are able to survive in acidic conditions below pH 3.0 and tolerate ethanol levels above 10% (v/v) and the sulfite concentrations used during wine processing or produced by yeast during AF (Lorentzen and Lucas, 2019). The capacity to compete in a harsh environment such as wine is due to elaborate survival strategies, and biofilm formation could be one of them. The term “biofilm” was firstly used by Costerton et al. (1978) who defined it “as matrix-enclosed bacterial populations adherent to each other and/or to surfaces or interfaces. This definition includes microbial aggregates and floccules and also adherent populations within the pore spaces of porous media.” Biofilm formation has been widely described in bacteria since in nature they rarely develop as planktonic cultures but exist as communities of sessile cells which grow as biofilms (Kolter, 2010; Berlanga and Guerrero, 2016). Biofilms offer bacteria several ecological and physiological advantages allowing metabolic cross-feeding, cell–cell interactions, and chemical and physical resistance (Berlanga and Guerrero, 2016). Those protective benefits of biofilms depend on their own structure and on the gene expression patterns of sessile cells (Burmølle et al., 2014; Martin et al., 2016).

Over the last decade, modern oenological approaches take advantage of biofilms to improve wine quality and offer new methods of technological control over industrial processes. These approaches are based on the evidence that planktonic and sessile cells show a different metabolism (Bastard et al., 2016; Berlanga and Guerrero, 2016; Coelho et al., 2019). Up to now, very little attention has been given to *O. oeni* biofilm formation. Bastard et al. (2016) showed that *O. oeni* cells organized in biofilms on oak chips were characterized by increased tolerance to wine stresses, malolactic activities, and the modification of wood volatile composition in the resulting wines. Similar data were obtained by Coelho et al. (2019) who observed that MLF occurred faster and with better reproducibility in sessile cells than in planktonic ones. Resulting wines showed a different volatilome, particularly when bacterial biofilms were present at the wood interface. Swiegers et al. (2005) suggested the involvement of some specific LAB pathways in volatile compound production, such as pathways involved in the metabolism of grape components or involved in the modification of yeast metabolites or release of flavor-active compounds (Hernandez-Orte et al., 2009). To date, only a couple of studies (Bastard et al., 2016; Coelho et al., 2019) have focused on the effect of sessile or on biofilm LAB on MLF and aroma profile of wine. In fact, the majority of studies evaluated the effect of biofilm formation on probiotic traits, spoilage potential, and biotechnological processes (wastewater treatment or acetic acid production) (Lebeer et al., 2007; Jones and Versalovic, 2009; Ramírez et al., 2015).

Thus, in this study nine *O. oeni* strains isolated from organic wines were studied for their adhesion ability on abiotic surfaces. Moreover, the impact of planktonic and biofilm detached cells on the MLF outcome and the aroma profile of resulting wine was studied.

## Materials and Methods

### Bacterial Strains and Growth Conditions

Nine *O. oeni* strains (MALOBACT-T1, ISO349, ISO351, ISO350, ISO359, ISO360, ISO358, ISO344, and ISO352) were previously isolated from Montepulciano d’Abruzzo organic wines undergoing spontaneous MLF as reported by Battistelli et al. (2020). These strains were technologically characterized and produced on demand and distributed to the wineries by Dalton Biotecnologie s.r.l. (Spoltore, Italy). The strains were routinely grown on modified MRS medium (mMRS) supplemented with fructose (5 g/l), malic acid (6 g/l), and cysteine (0.5 g/l) at pH 4.8 (Maicas et al., 1999) and incubated at 28°C under anaerobic conditions for 7 days. Strains were stored at −80°C in mMRS broth supplemented with glycerol (Sigma-Aldrich, Milan, Italy) at a final concentration of 20% v/v.

### Microbial Adhesion on Polystyrene Plates

Strains were grown in mMRS as previously described and then collected by centrifugation and suspended in a saline solution (8.5 g/l) to yield an initial concentration of 6 log CFU/ml. The ability to form biofilms was monitored growing the cells in flat-bottomed six-well cell culture plates (Costar, Corning, NY, United States) containing 5 ml of mMRS, or organic wine (0.8 g/l fermentable sugars, 14% v/v ethanol, pH 3.6, 2.01 g/l malic acid, 0.36 g/l lactic acid). Plates were incubated at 28°C under anaerobic conditions for 20 days. Uninoculated wells were used as negative controls. Media and planktonic cells were removed rinsing with saline solution. Sessile cells were collected pipetting up and down 10 times (Kubota et al., 2009). Both sessile and planktonic cells were serially diluted and plated on mMRS for viable count. Assays were performed in triplicate.

### Confocal Laser Scanning Microscopy

Biofilm formation in mMRS and organic wine was examined by confocal laser scanning microscopy (CLSM) using the Nikon A1-R confocal imaging system (Nikon Corp., Tokyo, Japan) controlled by Nikon NIS-Elements interface (Version 4.40, Nikon Corp., Tokyo, Japan). Bacterial fluorescent labeling was carried out using 1 μl of Hoechst 33342 dye 10 mg/ml (Thermo Fisher, Milan, Italy). All analyses were performed in triplicate.

### Malic Acid Degradation

Analyses were carried out using the organic red wine previously described. *Oenococcus oeni* strains were inoculated as planktonic cells or after development as biofilm (6 log CFU/ml). In particular, sessile cells were cultivated for 20 days in 20 ml of organic red wine in polystyrene plates. The 20-day-old biofilm was detached from the plate and inoculated into the wine. Similarly, planktonic cells were collected and used as inoculum. The malic acid degradation was determined according to Battistelli et al. (2020) by HPLC. After centrifugation of 10 ml for 10 min at 3,000 *g*, the samples were diluted five-fold with 22.5 mM H_2_SO_4_ solution (mobile phase) prior to injection of 10 μl into HPLC. The aroma profile was determined as reported below.

### Volatilome Analysis

Aroma compounds were determined by solid phase microextraction coupled with gas chromatography (GC-MS-SPME) using a GC-mass spectrometer Clarus SQ 8 S chromatograph/mass (GC-MS) spectrometer (Perkin Elmer, Boston, MA, United States) as previously described (Tofalo et al., 2016; Perpetuini et al., 2020). Volatile compounds were extracted in a 10-ml glass vial mixing 1 g NaCl with 5 ml of wine. A carboxen–polydimethylsiloxane-coated fiber (85 μm) (Sigma-Aldrich, St. Louis, MO, United States) was placed in the desorption chamber for 15 min, and the following program was applied: 50°C for 2 min; first ramp, 1°C min to 65°C; second ramp, 10°C min to 150°C (10 min hold); third ramp 10°C min to 200°C (1 min hold). Volatile compounds were presumptively identified comparing mass spectra of compounds with those contained in the National Institute for Standards and Technology database (NIST version 2005). Quantitative data were obtained by interpolation of relative peak areas on the basis of calibration graphs built by the analysis of synthetic wines containing known amounts of the analytes, as previously described (Gómez-Míguez et al., 2007). Pure hexanoic acid (0.1 M) (Sigma-Aldrich) was used as internal standard. All determinations were performed in triplicate.

### Statistical Analysis

Data were analyzed by means of Prism 7.0 program (GraphPad Software Inc., La Jolla, CA, United States). The data were analyzed using a two-way ANOVA, considering the media and the lifestyle as the two factors. A *post hoc* analysis was performed with multiple *t*-tests. A false discovery rate correction was implemented to prevent the false-positive occurrence. A level of *p* < 0.05 was considered statistically significant. Principal component analysis (PCA) based on the main volatile compounds released by strains was performed using XLSTAT 2014 software (Addinsoft, New York, NY, United States). For malic acid degradation, a repeated-measure ANOVA (RM-ANOVA) was performed, together with a *post hoc* analysis.

## Results

### *Oenococcus oeni* Adhesion Ability on Polystyrene Plates

**FIGURE 1:**
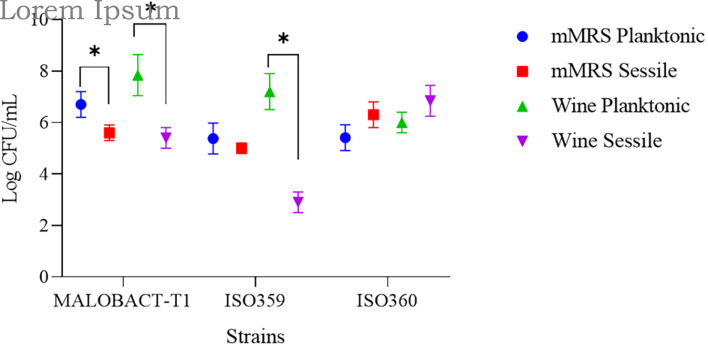
Planktonic and sessile cells determined by plate count in mMRS and organic wine. Results are represented as means ± SD of three experiments and expressed as log CFU/ml. The symbol ^∗^ denotes statistical differences (*p* < 0.05).

**FIGURE 2:**
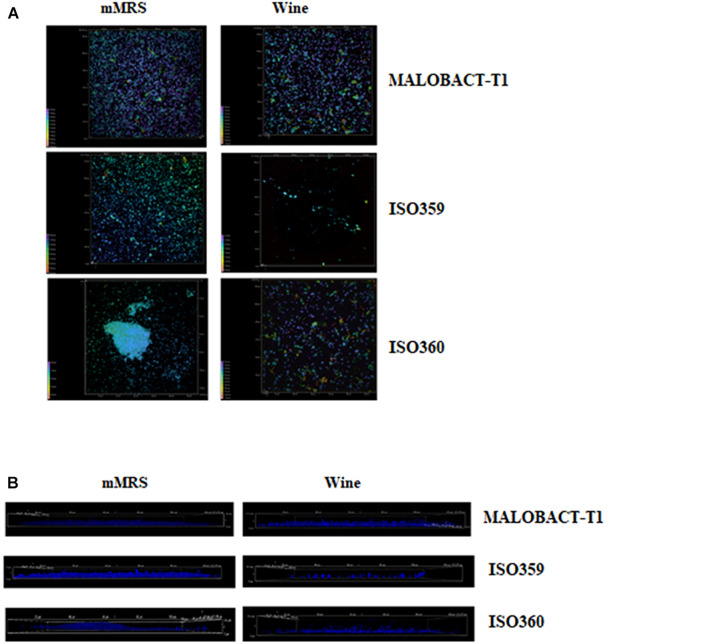
CLSM images of MALOBACT T1, ISO359, and ISO360 cultured in mMRS and wine. **(A)** ×100 3D images of strains. Colors from purple to red show the spatial organization of aggregates. Purple represents cells attached to the surface; red represents more distant cells. **(B)** ×100 3D images from the frontal view of strains.

In this study, nine strains of *O. oeni* were tested for their ability to adhere on polystyrene plates and only three strains—MALOBACT-T1, ISO359, ISO360—showed this capacity. The MALOBACT-T1 strain mainly developed as planktonic cells in both media, while the ISO359 strain mainly grew as planktonic cells in wine. The statistical analysis delivered a significant difference concerning the lifestyles; hence, a *post hoc* analysis associated with this factor was performed. The ISO360 strain showed similar values of plate counts in all conditions tested (Figure 1). MALOBACT-T1 showed the highest number of planktonic cells in both mMRS and organic wine. The highest plate count of sessile cells was detected for ISO360 strains with values of 6.3 ± 0.5 log CFU/ml and 6.8 ± 0.6 log CFU/ml in mMRS and organic wine, respectively. The ISO359 strain showed the lowest value of sessile cells in organic wine (2.9 ± 0.4 log CFU/ml) and mMRS (5 ± 0.2 log CFU/ml). In particular, a significant difference was observed between the number of planktonic and sessile cells of MALOBACT-T1 and ISO359 in organic wine. The strain-dependent adhesion ability was confirmed by CLSM. The adhesion ability of the MALOBACT-T1 strain was visualized in both conditions (Figures 2A,B). The ISO359 strain did not adhere when cultured in wine, in agreement with the low values of plate count, but sessile cells were detected in mMRS. ISO360 strains adhered in both conditions, and in mMRS, the formation of aggregates was observed.

### Malic Acid Degradation

**FIGURE 3:**
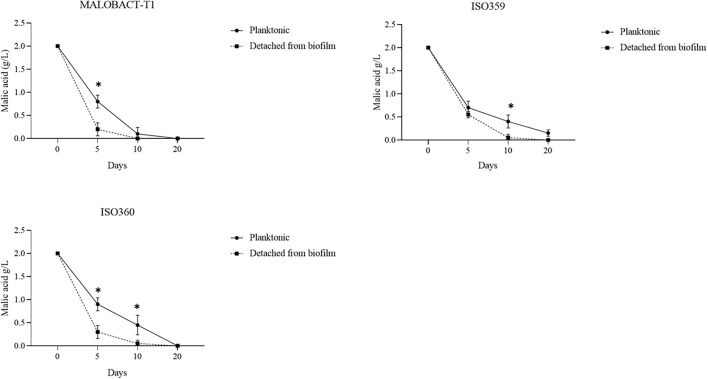
Comparison of malic acid degradation by tested strains. Strains were inoculated as planktonic cells or after development as biofilm. Error bars represent the standard deviation of three biological replicates. The symbol ^∗^ denotes statistical differences (*p* < 0.05).

The malic acid degradation ability of planktonic and biofilm-detached cells was monitored for 20 days. Planktonic cells of the ISO359 strain showed a residual concentration of malic acid of about 0.1 g/l. Detached cells degraded all the malic acid after 20 days and even in a shorter time (Figure 3).

The RM-ANOVA showed a significant difference for the malic acid degradation. The *post hoc* analysis revealed that after 5 days MALOBACT-T1 and ISO359 planktonic cells consumed 1.2 and 1.3 g/l of malic acid, respectively; ISO360 1.1 g/l. After 10 days, MALOBACT-T1 showed the highest malic acid degradation (1.9 g/l), and ISO359 and ISO360 strains showed values of 1.6 and 1.55 g/l, respectively. Detached cells had a better degradation ability. In fact, after 5 days MALOBACT-T1, ISO359, and ISO360 strains showed degradation values of 1.8, 1.45, and 1.7 g/l, respectively. After 10 days, MALOBACT-T1 consumed all the malic acid; the others showed a very low malic acid residual concentration (0.05 g/l) (Figure 3).

### Volatilome of Planktonic Cells

*Oenococcus oeni* shows several metabolic pathways and enzymes involved in volatile secondary compound production, including esters, higher alcohols, carbonyls, volatile fatty acids, and sulfur compounds (Bartowsky, 2005; Siebert et al., 2005). Planktonic cells produced a total of 42 compounds (Table 1). The total amounts of higher alcohols ranged from 25.41 mg/l (MALOBACT-T1) to 27.45 mg/l (ISO360), while esters varied from 86.54 mg/l (ISO359) to 88.21 mg/l (MALOBACT-T1) (Table 1). A strain–strain variation was detected. Esters were the main compounds produced. In particular, an increase in their content was observed compared to the initial wine. The main esters produced were acetic acid, 2-phenylethyl ester, decanoic acid, ethyl ester, octanoic acid, ethyl ester, and pentanoic acid, 2,2- dimethyl-, methyl ester, all related to fruity notes. Phenylethyl alcohol (sweet, fruity, and rose notes) was the main alcohol produced by *O. oeni* planktonic cells. In particular, a decrease of its levels compared to the initial wine was observed; however, its concentration was above the sensory threshold (14 μg/l) (Ferreira et al., 2000). Finally, *O. oeni* planktonic cells induced a reduction of organic acids.

**TABLE 1:** Main aroma compounds produced by planktonic and biofilm detached cells.

Aroma compounds	Initial wine	Planktonic cells			Biofilm-detached cells		
		MALOBACT-T1	ISO359	ISO360	MALOBACT-T1	ISO359	ISO360
**Higher alcohols**							
1-Hexanol	0.50 ± 0.08	0.3 ± 0.09	0.21 ± 0.08	0.11 ± 0.06	0.32 ± 0.1	0.12 ± 0.05	0.43 ± 0.18
1-Non-anol	0.02 ± 0.01	n.d.	n.d.	n.d.	n.d.	n.d.	n.d.
1-Dodecanol	0.03 ± 0.01	n.d.	0.03 ± 0.01	n.d.	n.d.	0.01 ± 0.01	0.02 ± 0.01
1-Octanol	0.20 ± 0.06	0.04 ± 0.02	n.d.	0.12 ± 0.04	0.03 ± 0.02	0.01 ± 0.01	0.03 ± 0.01
3-Methyl-1-pentanol	0.03 ± 0.01	0.02 ± 0.01	0.01 ± 0.01	n.d.	0.01 ± 0.01	n.d.	0.02 ± 0.01
5-Methyl-2-hexanol	0.07 ± 0.03	n.d.	n.d.	0.02 ± 0.01	n.d.	0.02 ± 0.01	n.d.
1-Butanol	0.70 ± 0.4	0.45 ± 0.15	0.54 ± 0.18	0.33 ± 0.1	0.25 ± 0.08	0.19 ± 0.08	0.34 ± 0.09
1-Butanol, 2-methyl-	5.76 ± 0.8	2.04 ± 0.5	1.89 ± 0.57	4.33 ± 0.9	0.54 ± 0.1	0.69 ± 0.2	1.44 ± 0.5
1-Butanol, 3-methyl-	2.87 ± 0.6	5.46 ± 0.8	3.83 ± 0.88	2.3 ± 0.65	2.54 ± 0.95	1.43 ± 0.55	5.12 ± 1.1
1-Pentanol	0.11 ± 0.3	0.43 ± 0.2	n.d.	n.d.	3.01 ± 0.7	n.d.	n.d.
2-Heptanol, 6-amino-2-methyl	n.d.	n.d.	n.d.	n.d.	1.21 ± 0.91	1.11 ± 0.45	1.02 ± 0.48
2-Methyl 1-propanol	0.65 ± 0.34	1.12 ± 0.66	0.34 ± 0.13	1.98 ± 0.88	0.89 ± 0.22	3.23 ± 0.88	1.78 ± 0.22
2-Pentanol	2.43 ± 0.53	0.93 ± 0.2	1.12 ± 0.34	0.92 ± 0.1	1.03 ± 0.36	6.09 ± 1.2	2.53 ± 0.76
3,4-Dimethyl-2-hexanol	n.d.	n.d.	n.d.	0.58 ± 0.12	n.d.	2.65 ± 0.9	0.81 ± 0.14
2,3-Butanediol	0.4 ± 0.1	0.51 ± 0.25	1.21 ± 0.42	1.43 ± 0.63	0.32 ± 0.1	n.d.	n.d.
Phenylethyl alcohol	19.54 ± 3.2	14.11 ± 2.5	17.03 ± 2.77	15.33 ± 3.22	14.01 ± 3.56	13.9 ± 2.11	16.12 ± 3.5
**TOT**	**33.31**	**25.41**	**26.21**	**27.45**	**24.16**	**29.45**	**29.66**
**Esters**							
3-Hydroxybutanoate	n.d.	0.94 ± 0.2	3.14 ± 0.9	2.12 ± 0.88	0.35 ± 0.13	0.78 ± 0.2	2.08 ± 0.65
Ethyl ethanoate	23.90 ± 2.2	21.37 ± 2.44	22.09 ± 3.53	21.87 ± 4.3	20.29 ± 3.1	21.24 ± 4.7	23.81 ± 4.1
1-Butanol, 2- methyl-, acetate	0.65 ± 0.21	n.d.	n.d.	n.d.	0.74 ± 0.2	n.d.	n.d.
1-Butanol, 3- methyl-, acetate	4.84 ± 0.46	8.98 ± 1.99	5 ± 0.91	9.12 ± 2.84	2.72 ± 0.9	1.77 ± 0.65	2.76 ± 0.9
1-Ethylpropyl octanoate	n.d.	0.48 ± 0.12	n.d.	n.d.	0.53 ± 0.12	n.d.	n.d.
Acetic acid, 2-phenylethyl ester	8.11 ± 1.67	9.8 ± 1.1	8.45 ± 1.94	8.3 ± 1.9	7.45 ± 1.3	6.34 ± 1.2	7.89 ± 1.4
Acetic acid, hexyl ester	n.d.	n.d.	n.d.	0.09 ± 0.01	0.86 ± 0.2	n.d.	n.d.
Benzoic acid, 2,6-bis[(trimethylsilyl) oxy]-, trimethylsilyl ester	n.d.	n.d.	n.d.	n.d.	0.75 ± 0.2	n.d.	n.d.
Butanedioic acid, diethyl ester	2.65 ± 0.9	5.77 ± 1.5	2.65 ± 1.2	3.88 ± 1.3	2.12 ± 0.9	n.d.	2.49 ± 0.4
Butanedioic acid, ethyl 3-methylbutyl ester	n.d.	0.36 ± 0.1	n.d.	n.d.	0.34 ± 0.1	n.d.	0.55 ± 0.12
Decanoic acid, ethyl ester	9.88 ± 2.24	10.23 ± 3.21	12.54 ± 4.3	11.54 ± 3.2	4.3 ± 0.6	7.9 ± 3.45	5.5 ± 1.33
Dodecanoic acid, ethyl ester	2.76 ± 0.4	1.68 ± 0.34	1.68 ± 0.84	1.37 ± 0.65	9.57 ± 2.3	8.24 ± 2.24	5.03 ± 2.01
E-11-Hexadecenoic acid, ethyl ester	n.d.	n.d.	n.d.	0.76 ± 0.2	0.64 ± 0.2	1.16 ± 0.12	1.99 ± 0.45
Ethyl 9,12-hexadecadienoate	1.11 ± 0.43	n.d.	n.d.	n.d.	n.d.	n.d.	0.98 ± 0.21
Ethyl 9-decenoate	8.29 ± 2.32	3.74 ± 1.4	3.87 ± 1.12	2.82 ± 1.12	n.d.	n.d.	n.d.
Hexadecanoic acid, ethyl ester	1.12 ± 0.4	0.54 ± 0.12	n.d.	n.d.	0.12 ± 0.1	0.91 ± 0.14	2.41 ± 0.87
Hexanedioic acid, monoethyl ester	n.d.	n.d.	n.d.	n.d.	0.34 ± 0.1	0.85 ± 0.13	0.21 ± 0.1
Hexanoic acid, ethyl ester	0.35 ± 0.1	2.56 ± 0.43	7.61 ± 2.83	3.77 ± 0.89	6.45 ± 1.35	1.92 ± 0.43	4.9 ± 1.98
Methyl 2-methylhexanoate	0.28 ± 0.1	0.38 ± 0.12	n.d.	1.67 ± 0.34	1.58 ± 0.6	0.69 ± 0.04	1.41 ± 0.62
Non-anoic acid, ethyl ester	0.11 ± 0.03	n.d.	n.d.	n.d.	0.96 ± 0.21	n.d.	n.d.
Octanoic acid, ethyl ester	7.07 ± 1.51	9.98 ± 2.45	8.34 ± 1.3	7.98 ± 1.98	3.2 ± 0.8	2.1 ± 0.53	3.3 ± 0.32
Pentadecanoic acid, 3-methylbutyl ester	n.d.	1.21 ± 0.8	n.d.	n.d.	0.32 ± 0.1	n.d.	n.d.
Pentanoic acid, 2,2- dimethyl-, methyl ester	6.31 ± 2.83	7.65 ± 1.67	8.9 ± 2.4	9.76 ± 2.4	2.4 ± 0.34	2 ± 0.3	2.3 ± 0.76
Pentanoic acid, 2,4-dimethyl-methyl ester	n.d.	0.42 ± 0.12	1.79 ± 0.5	1.54 ± 0.62	n.d.	0.81 ± 0.23	n.d.
Pentanoic acid, 4- methyl-, ethyl ester	2.45 ± 0.83	n.d.	0.03 ± 0.01	0.01 ± 0.01	0.06 ± 0.01	n.d.	0.09 ± 0.01
Ethyl lactate	n.d.	n.d.	n.d.	n.d.	4.09 ± 1.23	5.88 ± 1.22	8.91 ± 2.6
Propanoic acid, 2- hydroxy-, ethyl ester	n.d.	n.d.	n.d.	0.05 ± 0.01	n.d.	2.04 ± 0.8	2.01 ± 0.94
3-methylbut-1-ylmethanoate	4.65 ± 1.92	2.12 ± 0.68	0.45 ± 0.2	1.33 ± 0.45	1.77 ± 0.33	2.77 ± 0.34	0.34 ± 0.1
Ethyl phenylacetate	0.20 ± 0.08	n.d.	n.d.	n.d.	n.d.	n.d.	n.d.
**TOT**	**84.73**	**88.21**	**86.54**	**87.98**	**71.95**	**67.40**	**78.96**
**Organic acids**							
Hexanoic acid	0.29 ± 0.03	0.01 ± 0.01	n.d.	n.d.	0.81 ± 0.2	1.3 ± 0.1	n.d.
n-Decanoic acid	0.32 ± 0.04	0.11 ± 0.09	0.12 ± 0.08	0.22 ± 0.07	1.14 ± 0.34	1.41 ± 0.2	0.87 ± 0.1
Acetic acid	0.65 ± 0.1	0.08 ± 0.01	0.16 ± 0.06	0.45 ± 0.24	0.5 ± 0.1	n.d.	n.d.
Octanoic acid	0.63 ± 0.2	n.d.	0.12 ± 0.05	0.09 ± 0.01	1.85 ± 0.45	1.52 ± 0.3	2.65 ± 0.45
3-Methyl butanoic acid	0.23 ± 0.01	0.01 ± 0.01	0.12 ± 0.07	0.02 ± 0.01	n.d.	n.d.	n.d.
Propanoic acid	0.07 ± 0.04	n.d.	0.12 ± 0.07	n.d.	n.d.	n.d.	n.d.
**TOT**	**2.19**	**0.21**	**0.64**	**0.78**	**4.3**	**4.23**	**3.52**

*p < 0.05.*

*n.d., not detected.*

*Data are expressed as mg/l.*

### Volatilome of Detached Cells From Biofilm

To evaluate the influence of *O. oeni* lifestyle on the release of specific aroma compounds, the cells detached from biofilm were inoculated in wine as reported in Materials and Methods.

Cells detached from biofilms released a total of 47 compounds in wine (Table 1). Higher alcohols and esters varied from 24.16 mg/l (MALOBACT-T1) to 29.66 mg/l (ISO360) and from 67.40 mg/l (ISO359) to 78.96 mg/l (ISO360), respectively. Organic acid concentrations ranged from 3.52 mg/l (ISO360) to 4.3 mg/l (MALOBACT-T1) (Table 1). These cells showed a different metabolism compared to the planktonic ones. In fact, resulting wines were characterized by a reduction of higher alcohols compared to the initial wine, but their content was higher than that produced by planktonic cells. Moreover, a decrease of ester content and an increase of organic acid concentration were observed. Biofilm-detached cells induced a reduction of some esters such as ethyl 9-decenoate, decanoic acid, ethyl ester, octanoic acid, ethyl ester, and pentanoic acid, 2,2- dimethyl-, methyl ester. However, biofilm-detached cells produced some specific compounds which were absent in wines inoculated with planktonic cells, such as 2-heptanol 6-amino 2-methyl, ethyl ester, hexanedioic acid, monoethyl ester, and ethyl lactate, all related to fruity and floral notes. Finally, biofilm-detached cells induced an increase of organic acid content, suggesting that the genes involved in their metabolism are upregulated in this kind of cells.

### Principal Component Analysis

**FIGURE 4:**
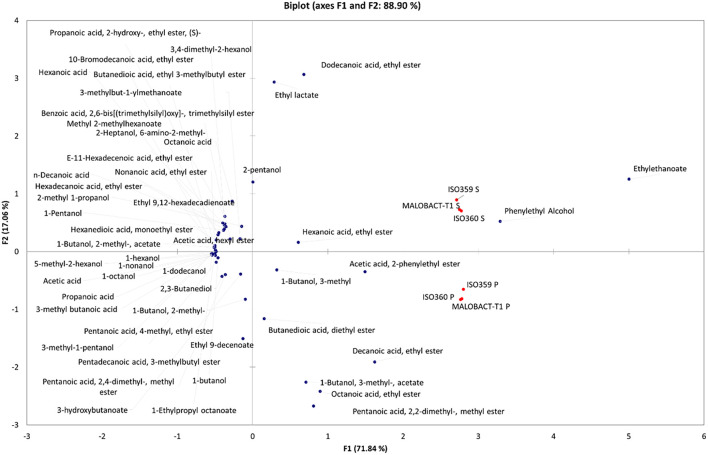
Principal component analysis (PCA) encompassing aroma compounds released in wines by planktonic and biofilm-detached cells of *O. oeni.* The biplot (score and loading) of the first two principal components showed 88.9% of the cumulative variance.

Principal component analysis allowed 88.9% of the total variance to be explained by the first two PCs (Figure 4), and 71.84% was attributable to PC1. Based on the distribution of samples, two groups were identified even if they were both in the right part of the PCA graph. The first one was made up of wines obtained with *O. oeni* inoculated as planktonic cells and were differentiated for acetic acid, 2-phenylethyl ester, 1-butanol, 3-methyl acetate, octanoic acid, ethyl ester, and pentanoic acid, 2,2-dimethyl, methyl ester. Wines obtained with *O. oeni* cells detached from biofilm belonged to the II group and were well differentiated for dodecanoic acid, ethyl ester, ethyl lactate, ethyl ethanoate, phenylethyl alcohol, hexanoic acid, and ethyl ester. Obtained data suggested that *O. oeni* lifestyle exerts an effect in the definition of wine volatilome, suggesting that it should be possible to modulate wine characteristics using different inoculum strategies.

## Discussion

### Biofilm Forming Potential of *Oenococcus oeni Strains*

Biofilm formation has been described in several bacterial species and represent the dominant mode of microbial existence (Costerton et al., 1995). The organization in biofilm has been described in *O. oeni* strains by other authors who observed the ability of this species to adhere on different materials, e.g., polystyrene, oak chips, and stainless steel surfaces (Bastard et al., 2016; Coelho et al., 2019). Tested strains showed a different behavior which may be a result of the inter-strain genomic variation observed in this species with up to 10% variation in protein-coding genes, including those predicted to be involved in sugar utilization and transport, exopolysaccharide biosynthesis, amino-acid biosynthesis, and natural competence (Borneman et al., 2012; Sternes and Borneman, 2016; Sternes et al., 2017). These strains could also be able to adhere to other materials, such as oak chips and stainless-steel surfaces which are generally used in winemaking or form aggregates with other bacterial/yeast species. Moreover, because of the inter-strain genomic variability observed within this species, it should be useful to perform further studies on the *O. oeni* population of different origins to highlight the distribution of adhesion abilities and evaluate their impact on wine characteristics.

The presence of aggregates observed by CLSM could explain the culturable sessile cell decay of the ISO359 strain. In the case of ISO360 regardless of the formation of aggregates, a high adhesion ability was observed. In this case, the formation of aggregates could simply induce an underestimation of culturable cells. In fact, as suggested by Pannella et al. (2020), the formation of bacterial cell aggregates could give rise to a single colony on plate, resulting in an underestimation of culturable cells. This is a widely observed phenomenon among both environmental and pathogenic species and can affect cell fitness (Trunk et al., 2018). If competition between aggregates and single cells is high, aggregates exhibit higher fitness, because cells at the top of aggregates have a better access to nutrients (Kragh et al., 2016). Moreover, aggregates may represent a protected mode of bacterial colonization of new niches in a hostile environment as could be the wine (Kragh et al., 2016). The lower number of sessile cells than planktonic ones could also be due to other factors including the shift to a viable but non-culturable state, resulting in being unable to grow in routine bacteriological culturing media, while maintaining their metabolic activity (Oliver, 2005). This condition has been described in several bacterial species and also in *O. oeni* strains (Millet and Lonvaud-Funel, 2000).

### *Oenococcus oeni* Lifestyle Modulates Malolactic Fermentation Outcome

It is well known that malate metabolism is a strain-dependent feature (Moreno-Arribas and Polo, 2005; Battistelli et al., 2020), and on the basis of obtained data, it seems that it is also influenced by *O. oeni* lifestyle. The strain-dependent variability observed in detached cells could be due to the kinetics of malate metabolism as a function of the growth stage in the biofilm. In fact, biofilms are made up of heterogeneous populations with local patterns of metabolic pathways. A positive effect of biofilm lifestyle on MLF was reported by Pannella et al. (2020) for *Lpb. plantarum*. These authors revealed that *Lpb. plantarum* sessile cells and cells detached from biofilm showed a similar metabolism and had an improved ability to degrade malic acid than planktonic cells. Similarly, Bastard et al. (2016) showed that *O. oeni* sessile cells performed complete MLF with a better kinetic than planktonic cells. Probably—as happens for other LAB—in *O. oeni* malate metabolism genes are upregulated under stressful conditions (Miller et al., 2011; Ma et al., 2018). In fact, the conversion of malate in lactate results both in ATP synthesis and in pH homeostasis (Konings, 2002; Beltramo et al., 2006; Miller et al., 2011). The different malic acid kinetics observed could be related also to the genetic variation between strains and a differential expression of genes involved in malate metabolism in sessile cells (Bon et al., 2009), suggesting a relationship between genome variation and efficient malate metabolism showing the occurrence of eight stress-responsive genes which could be associated with high MLF performance.

### *Oenococcus oeni* Lifestyle Modulates Aroma Profile of Wines

*Oenococcus oeni* strains tested in the study exerted a strain-dependent effect on wine volatilome in both lifestyles. Moreover, detached cells from biofilms and planktonic cells induced specific modifications of wine aroma profile. The strain-dependent behavior of *O. oeni* planktonic cells on esters’ content has been reported also by other authors (Matthews et al., 2004; Bartowsky, 2005; Francis and Newton, 2005; Brizuela et al., 2017, 2018). However, there is a great disagreement concerning this aspect. In fact, some studies reported an increase in this class of compounds (Delaquis et al., 2000; Costello et al., 2013; Brizuela et al., 2017, 2018), while in others, a decrease in the concentration of esters was reported (Du Plessis et al., 2002). The effect on ester content may be related to ester-synthesizing and -hydrolyzing activities. However, enzyme activities associated with ester biosynthesis are not well documented in *O. oeni* and some authors reported that this species shows hydrolyzing esterase activities (Davis et al., 1988; Matthews et al., 2006, 2007; Sumby et al., 2009, 2010; Pérez-Martín et al., 2013). Regarding the reduction of phenylethyl alcohol in wines inoculated with *O. oeni* planktonic cells, a similar tendency was observed by Brizuela et al. (2018) in Pinot noir wines, even if in that case the reduction was below the sensory threshold.

Obtained data revealed that detached cells from biofilm induced a decrease of some class of aroma compounds. This reduction may be related to a biofilm trapping activity and/or a differential expression of bacterial enzymatic activities, e.g., glycosidase or esterase, in biofilm-detached cells. Similarly, *O. oeni* strains adhered to oak chips can lead to a modulation of wine volatilome in terms of lowering the specific aroma compounds associated with the wood probably due to the presence of exopolysaccharide matrix of the biofilm (Bastard et al., 2016). The influence of *O. oeni* lifestyle on wine aroma composition has been poorly investigated. Recently, Bastard et al. (2016) suggested an alternative which exploits *O. oeni* cells organized in biofilms on oak chips to improve malolactic activity and modulate wood volatile composition in the resulting wines. The influence of sessile *O. oeni* cells on wine aroma has been shown also by Coelho et al. (2019) who revealed changes of concentrations in higher alcohols.

On the basis of data obtained in this study, it seems that the aroma profile of wines can be modulated by *O. oeni* lifestyle in a strain-dependent way. This evidence could be related to the genomic variation observed within this species. Bartowsky and Borneman (2011) observed a great variability in different *O. oeni* strains concerning the aroma-forming pathways and described the presence of additional glycosidases in some strains which could enhance red fruit–red berry aroma attributes to red wines. Therefore, the production of some specific compounds by detached biofilm cells may be related to the differential expression of some specific genes in these cells. However, further studies are necessary to verify this hypothesis.

This study highlighted that biofilm-detached cells of *O. oeni* showed an increased ability to degrade malic acid than planktonic cells and were able to modulate wine aroma both quantitatively and qualitatively. The application of new *O. oeni* inoculation strategies could represent a useful tool to develop new strategies to modulate wine aroma. Moreover, the inter-strain genetic variation of the *O. oeni* genome together with its lifestyle (planktonic vs. sessile) represent the potential of its exploitation to modulate MLF outcome and wine aroma development in order to develop novel wine styles. Further studies will be performed, at industrial level, to validate the role of biofilm-detached cells of *O. oeni* and eventually propose a new way to develop starter cultures.

## Data Availability Statement

The original contributions presented in the study are included in the article, further inquiries can be directed to the corresponding authors.

## Author Contributions

RT: conceptualization, investigation, writing—review and editing, and funding acquisition. GP: conceptualization, writing—original draft, writing—review and editing, and data analysis. NB, LV, and AR: methodology. CP and CZ: funding acquisition. GA: review and editing. All authors contributed to the article and approved the submitted version.

## Conflict of Interest

CZ is employed by Orsogna winery and Carlo Perla by Dalton Biotecnologie s.r.l. The remaining authors declare that the research was conducted in the absence of any commercial or financial relationships that could be construed as a potential conflict of interest.

## Publisher’s Note

All claims expressed in this article are solely those of the authors and do not necessarily represent those of their affiliated organizations, or those of the publisher, the editors and the reviewers. Any product that may be evaluated in this article, or claim that may be made by its manufacturer, is not guaranteed or endorsed by the publisher.
